# Dynamic Metabolomic Changes During Superficial Scald Development and 1-MCP Intervention in ‘Dangshansuli’ Pears

**DOI:** 10.3390/foods15040622

**Published:** 2026-02-09

**Authors:** Zhihong Feng, Jiaxin Chang, Zhenfeng Gao, Xinxian Zhang, Lixin Zhang

**Affiliations:** 1 College of Food Science and Engineering, Shanxi Agricultural University, Taiyuan 030031, China; zhihongfeng@sxau.edu.cn (Z.F.); 20233666@stu.sxau.edu.cn (J.C.); zhenfenggao@sxau.edu.cn (Z.G.); zhangxinxian@sxau.edu.cn (X.Z.); 2 Shanxi Center of Technology Innovation for Storage and Processing of Fruit and Vegetable, Taigu, Jinzhong 030801, China

**Keywords:** *Pyrus bretschneideri*, metabolic reprogramming, physiological disorder, untargeted metabolomics, phenylpropanoid biosynthesis

## Abstract

Superficial scald, a postharvest physiological disorder of pears, leads to substantial economic losses. However, the dynamic metabolic shifts that underpin symptom development and the extent of metabolic reorganization triggered by the ethylene inhibitor 1-methylcyclopropene (1-MCP) remain poorly understood. We combined physiological assessments with untargeted metabolomics to track superficial scald development and 1-MCP’s inhibitory effect in ‘Dangshansuli’ pears (*Pyrus bretschneideri*). In control fruit, disorder progression followed a trajectory of escalating physiological disruption, initiated by an ethylene burst and early oxidative stress, which advanced to membrane lipid peroxidation, glutathione (GSH) depletion, and dysregulated phenolic metabolism. By contrast, 1-MCP fundamentally diverted this trajectory by acting both as an initial ethylene antagonist and a sustained broad-spectrum metabolic regulator. It time-dependently reconfigured secondary metabolism away from the production of scald-promoting compounds (e.g., α-farnesene and phenolic acids) and toward the sustained synthesis of protective alkaloids and flavonoids, thereby enhancing cellular homeostasis and antioxidant capacity. KEGG and correlation network analyses confirmed that 1-MCP uncoupled the metabolic network from oxidative damage. Our findings indicate that 1-MCP is associated with a comprehensive metabolic reprogramming that shifts the fruit away from the scald development trajectory, providing a mechanistic foundation for developing metabolism-targeted strategies to control postharvest losses.

## 1. Introduction

Superficial scald is a prevalent postharvest physiological disorder in pome fruits, characterized by the development of brown or black patches on the fruit peel, typically emerging after prolonged cold storage and during subsequent shelf life [1]. While extensively studied in apples, this disorder also inflicts significant economic losses on pear production, especially in susceptible cultivars. The Chinese pear ‘Dangshansuli’ is a prime example of such high susceptibility, with potential losses exceeding 40% during long-term storage [2]. A primary feature in this and other susceptible cultivars is that visible scald symptoms often remain latent during cold storage, only manifesting after transfer to room-temperature shelf conditions. Various factors influence scald severity, including fruit maturity [3], light exposure [4], and storage atmosphere [5,6]. However, the underlying metabolic basis for this susceptibility remains unclear, obstructing the development of targeted solutions. Growing regulatory restrictions on synthetic antioxidants like diphenylamine (DPA) in some markets, alongside consumer demand for residue-free alternatives, highlights the urgent need for effective control strategies. However, developing such alternatives requires a deeper, systems-level understanding of the metabolic basis of scald susceptibility, which remains unclear for pears.

The etiology of superficial scald involves a complex interplay between ethylene action, oxidative stress, and specialized metabolism. A widely recognized initiating event is the ethylene-induced biosynthesis of α-farnesene, whose oxidation products, conjugated trienols (CTols), are cytotoxic. The role of secondary metabolites is pivotal in this process; while terpenoids like α-farnesene serve as precursors to browning, various phenolic compounds, particularly flavonoids, act as intrinsic antioxidants that can mitigate oxidative damage [7]. Conversely, the oxidation of phenolics by polyphenol oxidase (PPO) is the direct cause of tissue browning [8]. Accumulating evidence also suggests that specific alkaloids and other nitrogen-containing metabolites may participate in stress signaling or cellular homeostasis during cold storage [9]. The accumulation of CTols triggers membrane lipid peroxidation, compromising cellular integrity [1,2]. This process is exacerbated by the depletion of antioxidant defenses, such as glutathione (GSH), and the activation of PPO, initiating a self-perpetuating cycle of reactive oxygen species (ROS) accumulation, membrane degradation, and ultimately, enzymatic browning of the peel [7,8]. A key question, however, is whether this model, largely derived from apple studies, fully captures the primary triggers and progression dynamics in pears, particularly the early metabolic events that commit the fruit to the scald pathway.

Indeed, compared to apples, the specific metabolic determinants and progression dynamics of superficial scald in pears remain less defined. Previous metabolomic studies in apples have investigated volatile metabolic shifts during superficial scald development [10] and have identified key metabolic and transcriptional networks associated with susceptibility and resistance [11,12,13]. However, metabolic responses are often species- and cultivar-specific. In pears, while foundational work such as that of Giné-Bordonaba et al. [14] has begun to explore metabolic and transcriptional changes, a critical gap persists: the absence of a comprehensive, dynamic metabolomic profile that is directly linked to the visual stages of disorder progression. This gap impedes a systems-level understanding of how effective interventions like 1-MCP reconfigure the metabolic network to establish a resistant state [15,16].

With the declining use of DPA, research into alternative control strategies has intensified. Several promising options have emerged, including 1-MCP, phyto-squalane [17], lovastatin [18], and edible coatings [19,20]. Among these, 1-MCP is a highly effective ethylene action inhibitor. Mechanistically, 1-MCP operates through its high-affinity binding to ethylene receptors (*ETR1*, *ERS1*, etc.), with a binding affinity approximately 10 times greater than that of ethylene itself. This nearly irreversible binding blocks the ethylene signal transduction pathway, preventing the downstream expression of ripening-related and stress-responsive genes [21]. The kinetics of 1-MCP binding are characterized by rapid saturation of available receptors, which effectively halts the ethylene-mediated metabolic cascade during the early stages of cold storage [22]. Nonetheless, its precise role remains debated: is it primarily an ethylene antagonist, or does it function as a broader systemic metabolic regulator that orchestrates long-term reprogramming for resilience?

Untargeted metabolomics has become a powerful tool, allowing researchers to dissect the complex metabolic networks underlying superficial scald and 1-MCP response [14]. Recent studies in pears have linked the disorder to specific changes in compounds like waxes, phenolic metabolites, and volatile organic compounds [23,24,25]. To address the knowledge gaps regarding the dynamic metabolic trajectory of superficial scald and the systemic action of 1-MCP in pears, we designed a symptom-severity-based dynamic metabolomic study for ‘Dangshansuli’ pears. Instead of arbitrary time points, sampling was aligned with key biological transitions in superficial scald severity, from asymptomatic to mild and then severe symptoms, to directly link metabolic changes to pathological progression. This design establishes the scald progression in control fruit as a dynamic reference framework. By comparing this to the metabolic state of 1-MCP-treated fruit, which remains asymptomatic at matched sampling points, we aimed to directly identify the metabolic reprogramming responsible for inhibiting the disorder, focusing on the contrast between the pathological trajectory and the intervention-induced, disorder-suppressed state.

This study was therefore guided by two central hypotheses, framed as comparative questions: (1) How does the protective metabolic state orchestrated by 1-MCP differ fundamentally from the dysregulated metabolic trajectory of superficial scald development? (2) Does 1-MCP induce a comprehensive metabolic reprogramming that extends beyond ethylene antagonism, and what is the defining metabolic signature of this resistant state in ‘Dangshansuli’ pear? By integrating physiological assessments with dynamic metabolomics, we aimed to contrast the metabolic trajectory of scald development against the intervention-induced state, thereby elucidating the systemic reconfiguration that underpins 1-MCP-induced protection. This work aims to define the metabolic shifts that distinguish a scald-prone trajectory from a scald-suppressed state, providing a new framework for developing metabolism-based management strategies in pear.

## 2. Materials and Methods

### 2.1. Plant Material and Treatments

Fruits of ‘Dangshansuli’ pears (*Pyrus bretschneideri* Rehd.) were harvested at the commercial maturity phase (175 days post full bloom) from 30-year-old trees grafted onto *P. betulifolia* Bunge rootstocks in an orchard located in Taigu, China (37°26′ N, 112°32′ E). To ensure true biological independence, three separate orchard rows (spatially isolated, >50 m apart) were designated as three biological replicate blocks. From each block, uniformly sized and defect-free fruits (with a mean weight of 200 ± 15 g) were selected, yielding approximately 60 kg of fruit per block. Fruits from each block were transported to the laboratory within 2 h in insulated cartons.

Upon arrival, fruits from each block were randomized and divided into two groups: control (CK) and 1-MCP treatment (T). This resulted in six independent experimental units (3 blocks × 2 treatments). For the 1-MCP treatment group within each block, fruits were exposed to 1.0 μL·L^−1^ 1-MCP for 24 h at 20 °C in a sealed 500 L container [26]. The 1-MCP was generated from a powder formulation (Freshdoctor, Xianyang Xiqin Biotechnology Co., Ltd., Xianyang, China, 3.5% active ingredient, *w*/*w*). The corresponding control group from the same block was exposed to an equal volume of sterile water vapor under identical conditions, in a separate container.

After treatment, each of the six experimental units (e.g., Block1_CK, Block1_T, etc.) was handled separately throughout storage and sampling. Fruits were packaged in perforated polyethylene bags (0.03 mm thickness, ~20 kg fruit per bag) to maintain 90–95% relative humidity. All units were stored in separate plastic boxes and subjected to a slow cooling process mimicking commercial practice: temperature was initially set at 10.0 ± 0.5 °C and reduced by 1 °C every 3 days until reaching the final storage temperature of 0.0 ± 0.5 °C.

### 2.2. Superficial Scald Assessment and Sample Strategy

Fruits were stored at 0.0 ± 0.5 °C for 240 days, during which no superficial scald symptoms were observed. Subsequent symptom development occurred entirely during a shelf-life period at 20 °C in the dark.

To directly link metabolic changes to the biological progression of the disorder, we employed a symptom severity-based sampling strategy instead of using arbitrary time points. This approach was chosen to maximize the contrast between the pathological trajectory (in CK) and the intervention-induced, disorder-suppressed state (in T), focusing on metabolism most relevant to symptom expression and control. In this framework, the scald progression in control fruit served as the essential temporal and phenotypic reference for evaluating the efficacy and mechanism of the 1-MCP intervention.

The severity of superficial scald was assessed based on the percentage of affected peel area, following the visual grading system of Tian et al. [27] (detailed in Section 2.3.1). For the purpose of triggering metabolomic sampling at biologically distinct stages, we defined two key symptom thresholds based on this system:

Sampling of control (CK) fruits was triggered at key transitions in superficial scald severity, thereby defining the reference points for comparison:

Onset of Symptoms (CK1): When control fruits first exhibited clear, mild superficial scald symptoms, corresponding to the lower range of Grade I (affected area: 5–15% of the peel, versus ≤ 15% in the full grade definition). At this same time, asymptomatic peel samples were collected from 1-MCP-treated fruits (designated T1).

Severe Symptoms (CK2): When control fruits displayed severe superficial scald, corresponding to Grade III (affected area > 30% of the peel). Symptomatic peel was collected from control fruits (CK2). Concurrently, asymptomatic peel samples were collected from 1-MCP-treated fruits, which remained completely free of superficial scald (designated T2).

Additionally, baseline peel samples were collected from both groups immediately before the shelf-life period (day 0), when no symptoms were present (designated CK0 and T0).

Upon each sampling event, a defined number of fruits were taken from each of the six independent experimental units (3 blocks × 2 treatments). Specifically, from each unit, the peel was excised from 5 randomly selected fruits (regardless of symptom distribution for CK at CK1 and CK2). The peel disks from these 5 fruits within the same unit were pooled to form one composite biological sample for metabolomic and physiological analysis. Therefore, for each treatment and sampling point (e.g., CK1, T2), *n* = 3 independent composite biological samples were obtained, each derived from a distinct orchard block. In total, 5 fruits × 6 experimental units = 30 fruits were sampled per time point. All composite samples were immediately flash-frozen in liquid nitrogen and stored at −80 °C for subsequent analysis.

### 2.3. Measurement of Physiological Indices

#### 2.3.1. Determination of Superficial Scald Index (SSI)

The SSI was evaluated according to the method of Tian et al. [27]. Fruit were visually assessed and divided into 4 grades based on the area affected by superficial scald. Grade 0: no superficial scald; Grade I: superficial scald area ≤ 15%; Grade II: 15% < superficial scald area ≤ 30%; Grade III: superficial scald area > 30%. The SSI was calculated using the formula:

SSI (%) = [∑(Grade number × Number of fruit in that grade)/(3 × Total fruit number)] × 100(1)

#### 2.3.2. Determination of Ethylene Production Rate (EPR)

EPR was quantified as described by Tang et al. [28]. Three fruits per replicate were sealed in a 1.5 L container for 2 h at 20 °C. A 1 mL headspace gas sample was injected into a Shimadzu GC-14CK gas chromatograph (Shimadzu Corporation, Kyoto, Japan) equipped with a GDX-502 column (Restek Corporation, Bellefonte, PA, USA) and a flame ionization detector (FID). Operating conditions were as follows: column temperature 70 °C; detector temperature 110 °C; hydrogen pressure 0.7 kg cm^−2^; air pressure 0.7 kg cm^−2^; carrier gas (N_2_) pressure 1.0 kg cm^−2^. Results were expressed as µL·kg^−1^·h^−1^.

#### 2.3.3. Determination of α-Farnesene (AF) and CTols

The contents of α-farnesene and CTols in the peel were quantified based on a modified method of Anet [29]. Using a cork borer, 20 peel disks (10 mm diameter) were collected and placed into a 25 mL test tube. Subsequently, 10 mL of n-hexane was introduced, and oscillatory extraction was conducted at room temperature for 2 h. Following extraction, 2 mL of the supernatant was purified using a Florisil column (composed of magnesium silicate adsorbent; Sigma-Aldrich, St. Louis, MO, USA), followed by elution with n-hexane to a final volume of 5 mL. The absorbance of the resulting eluate was immediately measured at 232 nm using spectroscopy and used for quantitative analysis. Additionally, another 3 mL aliquot of the extract was taken to determine absorbance values at 281 nm and 290 nm for further calculation of component concentrations. The α-farnesene and CTols contents were calculated using the following equations, respectively:

α-farnesene content (nmol cm^−2^) = 2.5 A232/29,000lb × 107 (2)

CTols content (nmol cm^−2^) = (A281 − A290)/25,000lb × 107 (3)

where l = path length of the cuvette (1 cm); b = total sampling area of the pericarp disks (cm^2^).

#### 2.3.4. Determination of Malondialdehyde (MDA) and Hydrogen Peroxide (H_2_O_2_)

The MDA content in the tissue was quantified based on a modified version of the method established by Dhindsa et al. [30]. 1 g of frozen tissue sample was homogenized in 4 mL of ice-cold 5% (*w*/*v*) trichloroacetic acid, and the homogenate was centrifuged at 12,000× *g* for 20 min at 4 °C. Subsequently, 2 mL of the resulting supernatant was mixed with 3 mL of 0.67% thiobarbituric acid, and the reaction mixture was heated in a boiling water bath for 15 min. After the reaction, the mixture was immediately cooled on ice and then centrifuged again at 12,000× *g* for 10 min. The absorbance of the final supernatant was measured at 450 nm, 532 nm, and 600 nm using an Ultrospec 2000 spectrophotometer (Amersham Biosciences, Little Chalfont, UK), and these absorbance values were used for quantitative analysis. The MDA content was calculated using the following formula:

MDA content (nmol g^−1^) = 6.45 × (A532 − A600) − 0.56 × A450 (4)

Following the method of Loreto and Velikova [31] with slight modifications, H_2_O_2_ content was determined. Briefly, 2 g of pericarp tissue was homogenized in 5 mL of pre-cooled acetone. The homogenate was then centrifuged at 8000× *g* and 4 °C, and the resulting supernatant was collected and refrigerated for subsequent analysis. Reaction system: mix 1 mL of extract, 100 μL of 10% titanium tetrachloride hydrochloric acid solution, and 100 μL of concentrated ammonia, react for 5 min, then centrifuge at 8000× *g*, take the precipitate, repeatedly wash off the pigments with cold acetone, add 3 mL of 2 mol·L^−1^ sulfuric acid, and determine the OD value at 412 nm. The H_2_O_2_ content was quantified by referencing a pre-established standard calibration curve, which was constructed using H_2_O_2_ solutions of varying concentrations. The results were reported in µmol·g^−1^ FW.

#### 2.3.5. Determination of GSH and PPO

The GSH content and PPO activity were measured using an Ultrospec 2000 spectrophotometer (Amersham Biosciences, Little Chalfont, UK) following the methods of Cao et al. [32] with adaptations. The GSH content and PPO activity were calculated using the following equations:

GSH content (μmol·g^−1^) = (N × V_t_)/(V_s_ × W) (5)

PPO activity (U·g^−1^) = (ΔA × V_t_)/(0.01 × Δt × V_s_ × W) (6)

where N = amount of GSH (μmol) from the standard curve, V_t_ = total extract volume (mL), V_s_ = volume used for assay, W = sample fresh weight, ΔA = absorbance change per minute, Δt = reaction time (min), and one unit (U) of activity is defined as the amount causing a change of 0.01 in absorbance per minute.

### 2.4. Metabolite Extraction

Following homogenization in liquid nitrogen, each sample was mixed with 400 μL of a pre-chilled methanol/acetonitrile/water solution (4:4:2, *v*/*v*) and vortexed. The mixture was incubated at −20 °C for 60 min and subsequently centrifuged (14,000× *g*, 4 °C, 20 min). The resulting supernatant was collected and dried under vacuum. For LC-MS analysis, the residue was reconstituted in 100 μL of acetonitrile-water (1:1, *v*/*v*), followed by vortexing and centrifugation under the same conditions. Finally, 2 μL of the supernatant was injected for analysis.

### 2.5. Metabolomics Analysis

An untargeted metabolomics approach was employed to profile the metabolites.

#### 2.5.1. Chromatographic Conditions

Chromatographic separation was carried out on an ACQUITY UPLC BEH C18 column (100 mm × 2.1 mm, 1.7 μm; Waters, Milford, MA, USA) maintained at 40 °C. The flow rate was set at 0.3 mL/min, with mobile phase A consisting of water containing 0.1% formic acid and mobile phase B being acetonitrile. A gradient elution was applied under the following conditions: 5% B from 0 to 0.5 min, held at 5% B until 1.0 min, increased linearly from 5% to 100% B between 1.0 and 9.0 min, maintained at 100% B from 9.0 to 12.0 min, and returned to 5% B by 15.0 min. The injection volume was 5 μL. Samples were maintained at 4 °C in the auto-sampler. To minimize the impact of instrument signal fluctuations, samples were analyzed in a random order. Quality control (QC) samples were inserted at regular intervals throughout the sequence to monitor system stability and data reliability.

#### 2.5.2. Mass Spectrometric Conditions

Detection was performed using an electrospray ionization (ESI) source (integral to the Q-Exactive mass spectrometer; Thermo Fisher Scientific, Waltham, MA, USA) operating in both positive and negative ion modes. The primary data analysis and interpretation presented in this study are focused on the positive ion mode data. After UPLC separation, analyses were conducted on a Q-Exactive hybrid quadrupole-Orbitrap mass spectrometer (Thermo Fisher Scientific, Waltham, MA, USA). Key ion source settings included: ion source gas 1 (Gas 1): 60; ion source gas 2 (Gas 2): 60; curtain gas (CUR): 30; source temperature: 320 °C; and ion spray voltage floating (ISVF) at ±3500 V. Mass spectrometric acquisition parameters were defined as follows: full MS scan over *m*/*z* 80–1200; MS^2^ resolution: 17,500; full MS accumulation time set to 0.20 s/spectrum; MS/MS accumulation time: 0.05 s/spectrum. MS/MS spectra were gathered via an information-dependent acquisition (IDA) approach under high-sensitivity settings. The declustering potential (DP) was maintained at ±60 V, with a collision energy of 35 ± 15 eV. The IDA configuration specified: exclusion of isotopic ions within 4 Da; fragmentation of the six most intense ions per scanning cycle.

### 2.6. Data Processing and Metabolite Identification

The raw LC-MS/MS data files were processed using Compound Discoverer 3.0 (Thermo Fisher Scientific, Waltham, MA, USA) for peak detection, alignment, and normalization. To ensure data quality for subsequent statistical analysis, metabolic features with missing values greater than 50% across all samples were filtered out prior to identification. Additionally, background signals were subtracted by comparing sample data against blank solvent injections. Following these preprocessing steps, metabolite annotation was performed by matching the accurate mass (with a mass error tolerance < 25 ppm) and MS/MS spectra against a self-built database (provided by Nanjing Personal Gene Technology Co., Ltd., Nanjing, China) and public databases, including HMDB, METLIN, BioCyc, HFMDB, and LipidMaps. The confidence of metabolite identification was assessed according to established metabolomics reporting standards. For this untargeted study, the highest annotation level achieved for downstream biological interpretation (e.g., of specific alkaloids and flavonoids) was Level 2, which requires both accurate mass and MS/MS spectral match to reference libraries.

### 2.7. Statistical Analysis

All statistical analyses were performed with a significance level of *p* < 0.05. Quantitative data are presented as mean ± standard deviation (SD) based on three biological replicates.

#### 2.7.1. Analysis of Physiological Indices

Temporal changes within each treatment group were analyzed separately. Data from the control group (sampling points CK0, CK1, CK2) and the 1-MCP-treated group (T0, T1, T2) were each subjected to one-way analysis of variance (ANOVA). When ANOVA indicated significant differences, Tukey’s honestly significant difference (HSD) post hoc test was applied for multiple comparisons among time points within the same group. These analyses and the generation of corresponding bar charts were conducted using Origin 2021 software (OriginLab Corporation, Northampton, MA, USA).

#### 2.7.2. Analysis of Metabolomics Data

Systematic quality assurance and preprocessing were implemented prior to the statistical analysis of metabolomics data. The sample injection order was randomized to minimize time-dependent bias. Subsequently, total peak area normalization was applied to adjust for systemic variations in overall signal response, and metabolic features with a relative standard deviation (RSD) > 20% in quality control (QC) samples were removed. No additional algorithm-based signal or drift correction (e.g., LOESS, SVR) was applied.

The identification of differential metabolites employed a strategy combining univariate and multivariate analyses. For pairwise group comparisons (e.g., CK0 vs. CK1), differential metabolites were identified using a two-tailed Student’s *t*-test. To control the false discovery rate (FDR) in the context of multiple comparisons across nearly 1000 metabolic features, *p*-values from the *t*-tests were adjusted using the Benjamini–Hochberg procedure. Metabolites with a fold change ≥ 2.0 (up-regulated) or ≤0.5 (down-regulated) and an FDR-adjusted *p*-value < 0.05 were considered statistically significant. These results were visualized in volcano plots using R software (version 4.3.2). For multivariate analysis, principal component analysis (PCA) and orthogonal partial least squares-discriminant analysis (OPLS-DA) were performed on Pareto-scaled data using SIMCA-P (version 14.1, Umetrics, Umeå, Sweden). Model quality was assessed using the parameters R^2^Y (goodness of fit) and Q^2^ (predictive ability).

Based on the differentially abundant metabolites identified above, Visualization and functional interpretation were conducted to elucidate the biological significance of the data. Venn diagrams, pie charts, and heatmap bubble plots for secondary differential metabolites were generated using R (with the ‘ggplot2’ package, version 3.4.4) and the OmicStudio online tool (version 3.0; Shanghai Biotree Biomedical Technology Co., Ltd., Shanghai, China), respectively. Significant metabolites were subjected to KEGG pathway annotation and analysis, with results visualized as Sankey bubble plots via OmicStudio. Correlation analysis between key metabolic pathways and all physiological indices was performed to generate a correlation network diagram. Finally, key metabolites were integrated for mechanistic pathway analysis based on the KEGG database.

## 3. Results

### 3.1. Physiological Changes During Superficial Scald Development and in Response to 1-MCP

**Figure 1:**
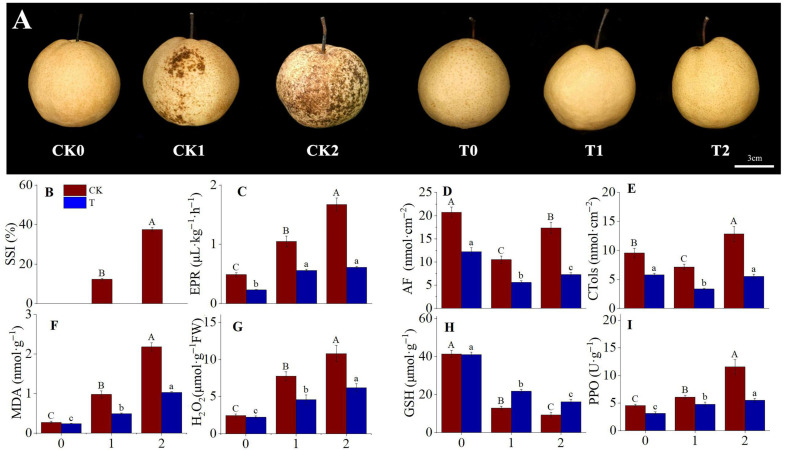
Changes in major physiological indicators during superficial scald development and its inhibition by 1-MCP in ‘Dangshansuli’ pear. (**A**) Fruit appearance at each sampling point: CK0 (asymptomatic control), CK1 (mild scald), CK2 (severe scald), T0 (asymptomatic 1-MCP), T1 (asymptomatic 1-MCP, matched with CK1), T2 (asymptomatic 1-MCP, matched with CK2), scale bar = 3 cm. Images are framed in red for control (CK) and blue for 1-MCP treatment (T). (**B**) Superficial Scald Index (SSI, %); (**C**) Ethylene Production Rate (EPR, µL·kg^−1^·h^−1^); (**D**) α-Farnesene (AF, nmol·cm^−2^); (**E**) Conjugated Trienes (CTols, nmol·cm^−2^); (**F**) Malondialdehyde (MDA, nmol·g^−1^); (**G**) Hydrogen Peroxide (H_2_O_2_, μmol·g^−1^); (**H**) Glutathione (GSH, µmol·g^−1^); (**I**) Polyphenol Oxidase (PPO, U·g^−1^). Data are mean ± SD (biological replicates, *n* = 3). Different uppercase letters (A–C) denote significant differences among sampling points within the control (CK) group, and lowercase letters (a–c) within the 1-MCP-treated group (*p* < 0.05, Tukey’s HSD). X-axis labels 0, 1, and 2 correspond to symptom-severity sampling points as defined in Section 2.2: asymptomatic baseline, mild scald onset, and severe scald, respectively.

To delineate the physiological dynamics associated with superficial scald, key indicators were tracked in control and 1-MCP-treated ‘Dangshansuli’ pears. In control fruits, scald development followed a trajectory of escalating disruption (Figure 1A,B), initiated by a marked ethylene surge (Figure 1C). This ethylene burst triggered α-farnesene metabolism (Figure 1D) and early oxidative stress. As symptoms progressed from mild (CK1) to severe (CK2), a critical acceleration occurred: oxidative markers (MDA, H_2_O_2_) increased more than 2-fold, while the antioxidant GSH was depleted by nearly 70% (Figure 1F–H). This non-linear escalation indicates a pivotal shift where cellular antioxidant defenses were overwhelmed, leading to irreversible membrane damage. Concurrently, the toxic oxidation product of α-farnesene, CTols, accumulated to 12.84 ± 0.90 nmol·cm^−2^ (Figure 1E), and PPO activity rose sharply to 11.54 ± 0.81 U·g^−1^ at CK2 (Figure 1I), creating the conditions for enzymatic browning.

In contrast, 1-MCP treatment effectively arrested this entire progression. The EPR in treated fruit (T2) was significantly suppressed (Figure 1C), which in turn inhibited both the synthesis and oxidation of α-farnesene (Figure 1D,E). Moreover, 1-MCP markedly alleviated oxidative stress, constraining the accumulation of MDA and H_2_O_2_ to 0.91 ± 0.06 nmol·g^−1^ and 5.88 ± 0.41 μmol·g^−1^, respectively (Figure 1F,G), sustaining GSH levels above 21.20 μmol·g^−1^ (Figure 1H), and limiting PPO activity to below 6.90 ± 0.48 U·g^−1^ (Figure 1I). Consequently, 1-MCP treatment completely prevented the development of superficial scald (Figure 1A,B).

### 3.2. Metabolic Changes in Response to Superficial Scald Development and 1-MCP Treatment

**Figure 2:**
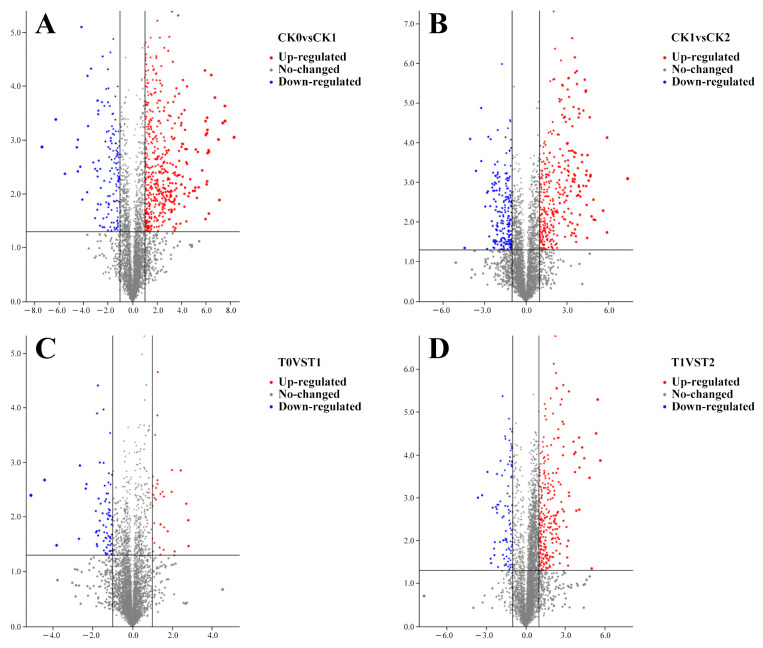
Volcano plots of differential metabolites (positive-ion mode) identified during superficial scald development and 1-MCP intervention. Metabolites with |log_2_FC| ≥ 1 and *p* < 0.05 were considered significantly altered. Red points: up-regulated metabolites (FC ≥ 2); blue points: down-regulated metabolites (FC ≤ 0.5); gray points: non-significant changes. The horizontal line represents the significance threshold (*p* < 0.05). (**A**) CK0 vs. CK1; (**B**) CK1 vs. CK2; (**C**) T0 vs. T1; (**D**) T1 vs. T2. Numbers in each plot indicate the count of up- and down-regulated metabolites.

Having established the physiological dynamics of superficial scald development, we employed untargeted UHPLC-MS/MS metabolomics to elucidate the underlying molecular basis and the protective mechanism of 1-MCP. A total of 967 metabolites were identified in the positive ion mode (Supplementary Table S1). Differential metabolites (defined as fold change (FC) ≥ 2 or ≤0.5, and *p* < 0.05) are presented in volcano plots (Figure 2A–D).

In control fruits, the number of differential metabolites increased with superficial scald severity, rising from 299 (204 up-regulated, 95 down-regulated) in the CK0 vs. CK1 comparison (asymptomatic to mildly symptomatic) to 325 (187 up-regulated, 138 down-regulated) in the CK1 vs. CK2 comparison (mild to severe symptoms) (Figure 2A,B). This trend indicates escalating metabolic dysregulation during disorder progression.

1-MCP treatment fundamentally altered this metabolic trajectory. In the early comparison (T0 vs. T1), coinciding with the onset of symptoms in controls, a smaller number of differential metabolites (160; 71 up-regulated, 89 down-regulated) was detected (Figure 2C). Strikingly, in the later comparison (T1 vs. T2), a period during which control fruits developed severe symptoms, the number of differential metabolites in 1-MCP-treated fruit surged to 368 (278 up-regulated, 90 down-regulated), despite the fruit remaining completely free of superficial scald (Figure 2D). This indicates that 1-MCP induces a profound and protective metabolic reorganization distinct from the disorder-associated dysregulation.

### 3.3. Contrasting Changes in Secondary Metabolism Induced by Superficial Scald Development and 1-MCP Treatment

**Figure 3:**
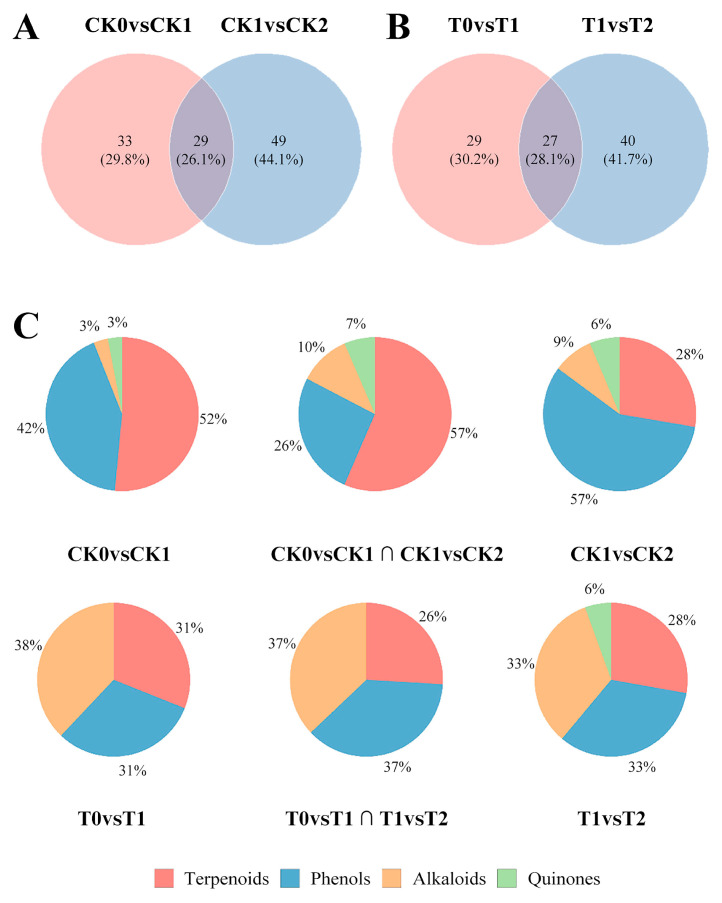
Differential distribution and class composition of secondary metabolites during superficial scald development and 1-MCP intervention. (**A**) Venn diagram for the control (CK) group, illustrating metabolites that were differentially abundant (fold change ≥ 2 or ≤0.5, *p* < 0.05) in the comparison between asymptomatic and mild scald stages (CK0 vs. CK1) and between mild and severe scald stages (CK1 vs. CK2). Each region represents metabolites unique to or shared between these comparisons. (**B**) Venn diagram for the 1-MCP-treated (T) group, showing differentially abundant metabolites in the comparisons between matched asymptomatic time points (T0 vs. T1 and T1 vs. T2). (**C**) Pie charts displaying the proportion of four major metabolite classes (terpenoids, phenolics, alkaloids, quinones) within each corresponding region of the Venn diagrams above (**A**,**B**).

Considering the direct association between phenolic oxidation and browning, our research centered on secondary metabolism. In the control fruit, the transition to severe superficial scald (from CK1 to CK2) was accompanied by a notable enrichment of specific phenolic compounds (28 out of 49 distinct metabolites; Figure 3A,C), which provided an ample supply of substrates for PPO-mediated browning.

In contrast, the fruit treated with 1-MCP displayed a distinct secondary metabolic pattern characterized by an enrichment of alkaloids. These alkaloids accounted for a significantly higher proportion (37.0–41.0%) of differential metabolites in all comparisons compared to the control group (Figure 3B,C). This finding implies that 1-MCP induces a shift towards a metabolic state enriched in alkaloids, which is inconsistent with the development of superficial scald.

### 3.4. Accumulation Patterns of Differential Secondary Metabolites in Control and 1-MCP Fruit

**Figure 4:**
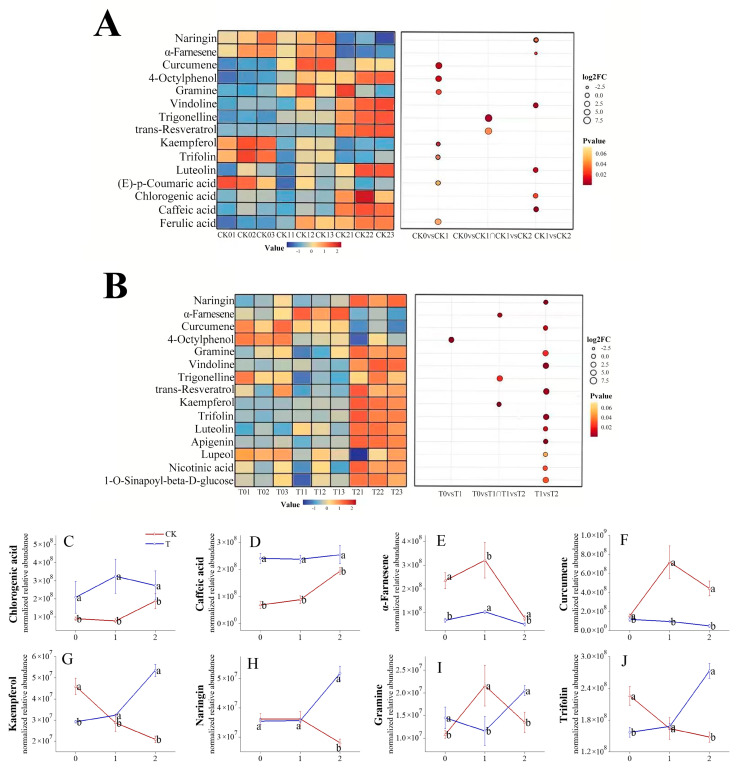
Distinct accumulation patterns of key secondary metabolites during superficial scald development and 1-MCP intervention. Hierarchical clustering heatmaps of 15 key differential metabolites are shown for the control (**A**) and 1-MCP-treated (**B**) groups. Each row represents a metabolite, and each column represents a symptom-severity sampling point (0, 1, 2, as defined in Section 2.2). The color scale from blue to red indicates the normalized relative abundance (z-score) of each metabolite, with blue representing lower levels and red representing higher levels relative to the mean across samples. Superimposed on the heatmap, bubbles encode differential abundance: bubble size is scaled to the magnitude of the log_2_ fold change (log_2_FC), with larger bubbles representing more substantial changes in abundance; bubble color represents the statistical significance (*p*-value), with the gradient from yellow to red indicating increasing significance (i.e., a decreasing *p*-value). Dynamic trajectories of eight representative metabolites across the sampling sequence are presented in panels (**C**–**J**): Chlorogenic acid (**C**), Caffeic acid (**D**), α-Farnesene (**E**), Curcumene (**F**), Kaempferol (**G**), Naringin (**H**), Gramine (**I**), and Trifolin (**J**). Data are presented as mean ± SE (*n* = 3). Red lines with triangles: control group (CK); blue lines with circles: 1-MCP-treated group (T). Different lowercase letters (a,b) denote significant differences among sampling points (*p* < 0.05, Tukey’s HSD). X-axis labels 0, 1, and 2 correspond to symptom-severity sampling points as defined in Section 2.2: asymptomatic baseline, mild scald onset, and severe scald, respectively.

To delineate the metabolic alterations during the development of superficial scald, hierarchical clustering analysis of significant differential metabolites was conducted (Supplementary Figure S1). The analysis identified 15 key metabolites per group (Figure 4A,B), 11 of which were shared between the control (CK) and 1-MCP-treated fruit, with the remaining 4 being unique to each group, demonstrating distinct metabolite accumulation patterns.

The heatmap revealed distinctly different accumulation patterns between control and 1-MCP-treated fruit (Figure 4A,B). In the control fruit, several phenolic acids (e.g., chlorogenic acid, CGA) and the phenolic compound 4-octylphenol exhibited an accumulation trend across the sampling points (CK0 → CK1 → CK2) as the severity of superficial scald increased, whereas key flavonoids such as naringin and kaempferol showed a decline. At CK2, the level of α-farnesene decreased, which is consistent with oxidative depletion, while the terpenoid curcumene increased. Alkaloids such as gramine and vindoline were also up-regulated. In contrast, in 1-MCP-treated fruits, the accumulation patterns showed clear differences. Across the storage period (T0 → T1 → T2), 1-MCP treatment sustained or increased the levels of flavonoids (e.g., naringin, kaempferol) and suppressed the rise in terpenoids associated with scald (e.g., curcumene). Moreover, four distinct metabolites, including the flavonoid apigenin and the triterpenoid lupeol, were identified only in this group, with their levels remaining stable or increasing. Alkaloids such as gramine and vindoline also demonstrated enhanced accumulation under 1-MCP treatment.

To complement the static view from the clustering analysis with dynamic resolution, we traced eight representative metabolites across the sequential sampling points (0 → 1 → 2) (Figure 4C–J). This dynamic analysis confirmed the patterns seen in the heatmap and provided detailed insight into the timing of changes. The trajectories for metabolites linked to browning, such as CGA and caffeic acid, rose in controls but remained significantly lower in 1-MCP-treated fruit, despite a slight increase at the final time point (T2). Notably, their later accumulation in 1-MCP fruit did not lead to browning. α-Farnesene peaked and then collapsed in controls, whereas 1-MCP maintained low levels. Curcumene surged only in controls at the middle stage, and this increase was absent in 1-MCP fruit. For antioxidant-associated flavonoids and alkaloids, a steady decline was observed in controls, but their levels rebounded in 1-MCP fruit after the first storage period.

In summary, the results from both the clustering heatmap and the dynamic trajectories demonstrate that 1-MCP treatment fundamentally alters the accumulation patterns of secondary metabolites. It not only reduces compounds associated with browning and oxidation but also promotes and maintains the accumulation of protective flavonoids and alkaloids throughout storage.

### 3.5. KEGG Enrichment of Pathways Involved in Superficial Scald Development and 1-MCP Inhibition

**Figure 5:**
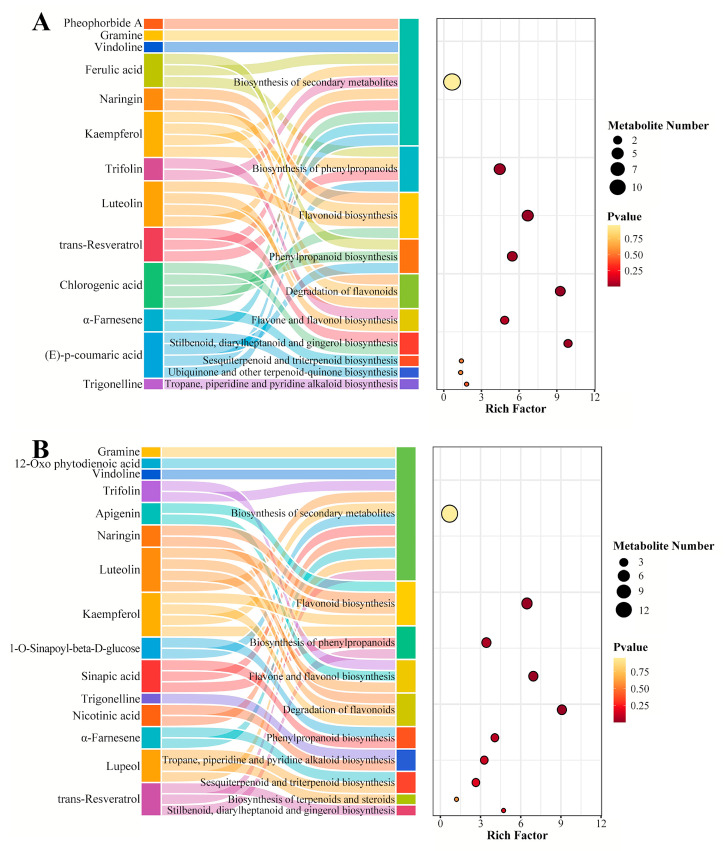
KEGG pathway enrichment analysis visualized using Sankey-bubble plots. These plots integrate two layers of information: bubble size represents the number of differentially abundant metabolites mapped to a given pathway; bubble color corresponds to the pathway enrichment factor (a measure of enrichment significance, with warmer colors indicating higher significance). The flow lines (Sankey links) between bubbles illustrate the shared metabolites connecting different enriched pathways. (**A**) Pathways enriched during superficial scald development in the control group (CK). (**B**) Pathways enriched in the 1-MCP-treated group (T). Only pathways meeting a significance threshold (corrected *p*-value < 0.05) are displayed.

To functionally interpret the metabolic alterations and identify the key pathways rewired by 1-MCP, we performed KEGG pathway enrichment analysis (Figure 5). Nine common metabolic pathways were found in both the control and 1-MCP-treated fruits, such as phenylpropanoid biosynthesis (map00940), flavonoid biosynthesis (map00941), and tropane, piperidine and pyridine alkaloid biosynthesis (map00960). However, the specific metabolites in these pathways were noticeably different.

For example, Flavonoid biosynthesis (map00941) was enriched in both groups. The control fruits mainly contained CGA, kaempferol, naringin, and luteolin, which were their characteristic components. The 1-MCP-treated fruits primarily had kaempferol, apigenin, luteolin, and naringin, forming the distinctive metabolites of this group. Notably, the control fruits specifically activated the ubiquinone and other terpenoid-quinone biosynthesis pathway (map00130), which was characterized by (E)-p-coumaric acid. The 1-MCP-treated fruits uniquely enhanced the biosynthesis of terpenoids and steroids (map01062), with lupeol as its featured metabolite.

### 3.6. Correlation Networks Among Physiological Indices and Metabolic Pathways in Superficial Scald Development and 1-MCP Inhibition

**Figure 6:**
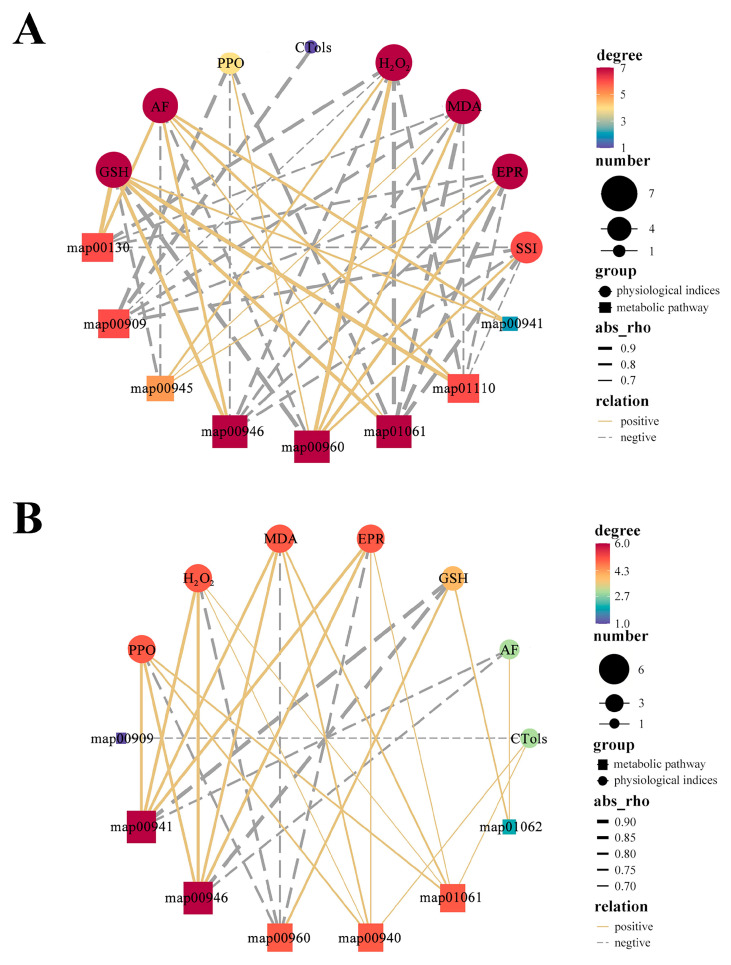
Pearson correlation networks integrating physiological indicators and key metabolic pathways during scald development and 1-MCP intervention. (**A**) Control group (CK); (**B**) 1-MCP-treated group (T). Circular nodes: physiological indicators; square nodes: KEGG metabolic pathways. Node color represents betweenness centrality, mapped to a color gradient from purple (low) to red (high). Node size is proportional to its degree centrality (i.e., the number of connections a node has), and the color intensity (darker shades) reflects betweenness centrality (higher importance in network connectivity). Solid yellow lines: significant positive correlations (r > 0.1, *p* < 0.05); dashed gray lines: significant negative correlations (r < −0.1, *p* < 0.05). Only correlations with |r| > 0.1 are shown. Line thickness corresponds to the absolute value of the Pearson’s correlation coefficient (|r|), with thicker lines indicating stronger correlations. Abbreviations for physiological indicators in the network: Ethylene Production Rate (EPR), α-Farnesene (AF), Conjugated Trienes (CTols), Malondialdehyde (MDA), Hydrogen Peroxide (H_2_O_2_), Glutathione (GSH), Polyphenol Oxidase (PPO), Superficial Scald Index (SSI). Abbreviations for metabolic pathways in the network: map00130 (ubiquinone and other terpenoid-quinone biosynthesis pathway), map00909 (Sesquiterpenoid and triterpenoid biosynthesis), map00940 (phenylpropanoid biosynthesis), map00941 (flavonoid biosynthesis), map00945 (stilbenoid, diarylheptanoid and gingerol biosynthesis), map00946 (Degradation of flavonoids), map00960 (tropane, piperidine and pyridine alkaloid biosynthesis), map01061 (biosynthesis of phenylpropanoids), map01062 (biosynthesis of terpenoids and steroids), map01110 (biosynthesis of secondary metabolites).

To gain a systems-level understanding of how metabolic pathways interact with physiological outcomes, we constructed a Pearson’s correlation network (Figure 6), with only associations characterized by |cor| > 0.1 and *p* < 0.05 being retained.

In control fruit (Figure 6A), core pathways like Degradation of flavonoids (map00946), Tropane, piperidine and pyridine alkaloid biosynthesis (map00960), and especially the overarching Biosynthesis of phenylpropanoids (map01061) showed strong correlations with EPR and ROS-related physiological indices (GSH, α-farnesene, MDA, H_2_O_2_), indicating that superficial scald development is driven by a system-wide rewiring of this network.

In the 1-MCP network (Figure 6B), the correlations between these key pathways (primarily Flavonoid biosynthesis map00941 and Degradation of flavonoid map00946) and injury markers (PPO, MDA, H_2_O_2_, EPR) were significantly attenuated, and the centrality of physiological indices was markedly reduced, reflecting a successful decoupling of metabolic activity from disorder symptomatology.

### 3.7. Integrated Metabolic Network Underlying Superficial Scald Development and 1-MCP Inhibition

**Figure 7:**
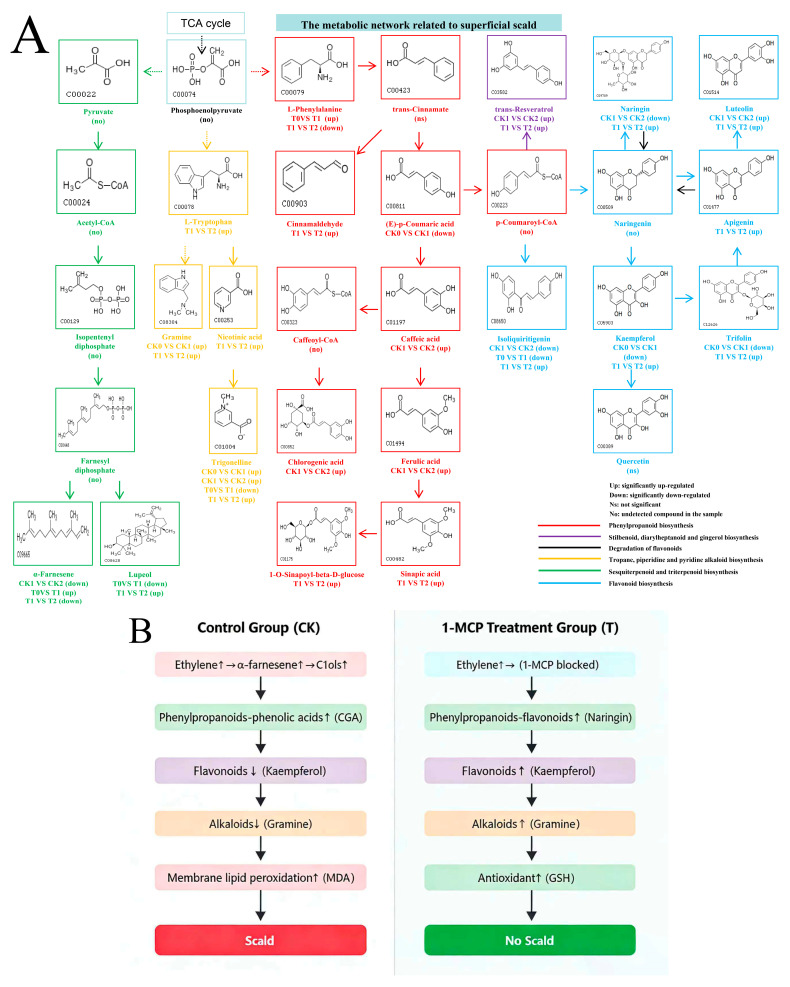
Proposed mechanistic framework of metabolic network shifts associated with superficial scald development and 1-MCP inhibition. (**A**) Metabolic pathway map comparing control (CK2) and 1-MCP-treated (T2) fruit. Metabolite boxes are color-coded by pathway: phenylpropanoid biosynthesis (red), stilbenoid, diarylheptanoid and gingerol biosynthesis (purple), degradation of flavonoid (black), tropane, piperidine and pyridine alkaloid biosynthesis (orange), sesquiterpenoid and triterpenoid biosynthesis (green), flavonoid biosynthesis (blue). Labels indicate regulation status: Up (significantly up-regulated in T2 vs. CK2), Down (significantly down-regulated), Ns (not significant), No (not detected). Arrows denote proposed metabolic fluxes. (**B**) Schematic trajectory contrasting scald development (left) and 1-MCP-induced protection (right). Key events include ethylene burst, α-farnesene oxidation, phenolic accumulation, flavonoid/alkaloid synthesis, and oxidative stress outcomes. The small arrows (↑) and (↓) indicate up-regulation and down-regulation of the depicted processes, respectively.

To integrate the physiological and metabolomic findings, a comprehensive model was constructed to visualize the metabolic shifts associated with the two contrasting states (Figure 7).

First, an expansive map details the reconfiguration of metabolites across six interconnected pathways between the control and 1-MCP-treated fruit at the final sampling point (CK2 vs. T2) (Figure 7A). In control fruit, the network showed a decline in flavonoid biosynthesis metabolites (e.g., naringin, kaempferol, trifolin) and an accumulation of specific phenolic acids (caffeic, ferulic, CGA) and stilbenoids (trans-resveratrol). Concurrently, sesquiterpenoid biosynthesis (α-farnesene) declined. In contrast, 1-MCP treatment was associated with an up-regulation of flavonoid biosynthesis, enhanced accumulation of alkaloids (L-tryptophan, gramine, trigonelline) and phenolic esters, and a shift in terpenoid metabolism toward compounds such as lupeol.

Second, a comparative trajectory model illustrates the sequence of events linked to each physiological outcome (Figure 7B). The schematic contrasts a loop in control fruit, initiated by an ethylene burst and involving α-farnesene oxidation, phenolic acid accumulation, and declines in flavonoids and alkaloids, culminating in membrane lipid peroxidation and scald. It also depicts an alternative pathway in 1-MCP-treated fruit, beginning with the blockade of ethylene perception, and is associated with enhanced synthesis of flavonoids and alkaloids alongside maintained antioxidant capacity.

In summary, by progressing from granular mechanistic insights to a streamlined causal narrative, this integrated model definitively characterizes 1-MCP’s mode of action. It functions not merely as an ethylene inhibitor but as a master regulator of metabolic reprogramming. Its efficacy is achieved through a tripartite mechanism: (1) blocking the initial trigger, (2) redirecting metabolic flux toward protective compound synthesis, and (3) fortifying cellular homeostasis. This comprehensive reprogramming diverts the fruit from a scald-conducive pathological trajectory onto a stable, stress-resilient trajectory. Our model thereby provides a robust mechanistic framework for developing next-generation, metabolism-targeted postharvest preservation strategies.

## 4. Discussion

### 4.1. The Dynamic Physiological and Metabolic Trajectory of Superficial Scald Development

It is important to note that the metabolic trajectory observed in control fruit (CK0 → CK1 → CK2) integrates both general postharvest senescence processes and the specific pathology of superficial scald development. Our sampling strategy, synchronized with scald severity, was designed to capture changes most relevant to the disorder’s expression. While some of the early shifts (e.g., the initial ethylene burst) may be common to ripening/senescence, the non-linear escalation of oxidative damage (e.g., GSH depletion, MDA surge) and the specific enrichment of browning-prone phenolic compounds at CK2 are strongly correlated with and characteristic of the transition to severe scald, distinguishing this trajectory from normal aging.

Our first hypothesis sought to define how the metabolic state in control fruit changes during scald development. By integrating dynamic metabolomic profiles with symptom severity, we mapped the metabolic trajectory of superficial scald development in ‘Dangshansuli’ pears. This trajectory progresses from an initial state of ethylene-driven stress to a state of severe oxidative and metabolic collapse. The observation that key damage markers (MDA, H_2_O_2_) and PPO activity increased sharply, while the rise in ethylene and α-farnesene was less pronounced during the transition from mild (CK1) to severe (CK2) symptoms highlights a critical inflection point within this continuum, consistent with the concept of antioxidant capacity being exceeded. This observed progression is consistent with previous reports in pears [2,21,33] and apples [34]. Our metabolomic results provide molecular evidence for this deterioration, showing that the fruit’s progressive loss of physiological integrity was linked to an accumulation of phenolic compounds (Figure 3A,C). This shift in the metabolic profile supplied abundant substrates for the rise in PPO activity (Figure 1I), directly linking elevated oxidative stress to peel browning, a process also documented in apples [35]. Having characterized this pathological metabolic trajectory, we next examined how 1-MCP treatment reprograms metabolism to achieve protection.

### 4.2. 1-MCP as a Master Regulator of Metabolic Reprogramming

Our second hypothesis questioned whether 1-MCP induces a comprehensive metabolic reprogramming beyond ethylene antagonism. The results support this view, indicating that 1-MCP orchestrates a sequential and broad reconfiguration of metabolism. Initially, it acts as an ethylene antagonist, but subsequently assumes a broader role as a central coordinator of metabolic resource allocation. This dynamic process, beginning with ethylene antagonism and progressing to a profound reorganization of secondary metabolism, highlights its role as a broad inducer of metabolic reorganization beyond the ethylene pathway, a theme consistent with but extending observations in apples [35]. While this broader regulatory role aligns with reports in apples where 1-MCP modulates phenolic and volatile pathways [35], the specific metabolic endpoints differ markedly. In apples, protection is frequently associated with sustained levels of certain flavonoids (e.g., epicatechin) and a reduction in specific phenolic acids [35]. In contrast, our study in ‘Dangshansuli’ pear reveals a pronounced and continuous enrichment of alkaloids (e.g., gramine, vindoline) as a hallmark of the protected state (Figure 4). This comparison suggests that while 1-MCP consistently acts as a master metabolic switch, the specific protective metabolome it orchestrates is likely cultivar- and species-dependent.

Our time-resolved analysis shows that 1-MCP triggers a temporally coordinated strategic shift. This temporal coordination is evident in the early-phase suppression of ethylene signaling and the initial oxidative burst, followed by the sustained, late-phase promotion of defense compound synthesis, such as alkaloids and flavonoids. This temporal shift in the number and class of differential metabolites (Figure 2 and Figure 3) underscores the “strategic” nature of the 1-MCP-induced response: it first blocks the initiating trigger and then actively builds a sustained defensive metabolism.

The integrated model derived from our data (Figure 7) propels this understanding from observation to a mechanistic hypothesis. It depicts 1-MCP’s mode of action not merely as an ethylene inhibitor but as associated with a broad metabolic reprogramming. The efficacy of 1-MCP, as suggested by the integrated model, can be conceptualized as involving a tripartite shift: (1) blocking the initial ethylene trigger, (2) redirecting metabolic flux away from scald-promoting pathways (e.g., specific phenolic acids, α-farnesene oxidation) and toward the synthesis of protective compounds (alkaloids, flavonoids, lupeol), and (3) thereby fortifying cellular homeostasis. This comprehensive reprogramming, as depicted in the comparative trajectory (Figure 7B), is associated with a diversion of the fruit from a scald-conducive pathological loop onto a stable, stress-resilient pathway. Our model thereby provides a testable mechanistic framework for developing next-generation, metabolism-targeted postharvest preservation strategies.

This expanded regulatory role is reflected in the distinct ways 1-MCP reprograms secondary metabolism as the disorder advances. By effectively curbing stress-induced alterations in secondary metabolism and redirecting metabolic flux, 1-MCP was associated with the synthesis of protective alkaloids (Figure 3C). Crucially, this remodeling of the secondary metabolome appears pivotal to 1-MCP’s success, as it coincides with a shift away from the production of browning-promoting phenolics toward the accumulation of defense compounds. Overall, our finding aligns with the emerging concept that inducing a protective, defense-oriented metabolic state is a key mechanism for enhancing postharvest resilience, as supported by studies in apples [34] and kiwifruits [36].

Notably, the continuous accumulation of alkaloids is a major factor behind this metabolic reprogramming. Beyond mere correlation, emerging evidence suggests alkaloids can function as antioxidants or signaling molecules in plant stress responses [37,38]. Building on this, we hypothesize that in our system, these alkaloids (e.g., gramine) may play a direct protective role, potentially acting as antioxidants mitigating oxidative stress, or as signaling molecules priming the fruit’s defense system. The results suggest that 1-MCP functions not only as an ethylene inhibitor but also as a broad-spectrum regulator that orchestrates a comprehensive anti-scald metabolic program, wherein the sustained synthesis of alkaloids represents a critical, actively deployed defense component worthy of future functional validation.

### 4.3. A Protective Metabolic Signature: Alkaloid and Flavonoid Enrichment

The comparative framework of our study directly addressed the second part of our central hypothesis, which sought to define the metabolic signature of the 1-MCP-induced resistant state. Profiling metabolites in relation to superficial scald severity enabled us to distinguish causal metabolic drivers from downstream consequences of the disorder. 1-MCP fundamentally reconfigured these severity-dependent accumulation patterns, as most prominently evidenced by the persistent suppression of α-farnesene (confirming a successful upstream blockade) and the concurrent, continuous accumulation of alkaloids and flavonoids in fruit without any symptoms (Figure 4). The notable enrichment of alkaloids under 1-MCP treatment, which accounted for a significantly larger proportion of the differential secondary metabolome compared to the control group (Figure 3B,C), is identified as a defining functional signature of the superficial scald-suppressive state.

This alkaloid/flavonoid-centric signature in ‘Dangshansuli’ pear presents a compelling contrast to established models in other fruits. The collective transition from a superficial scald-promoting profile (dominated by phenolic/quinone) to a superficial scald-suppressing profile (enriched in alkaloid/flavonoid) represents a successful metabolic rewiring away from damage-execution pathways and toward defense preparedness. In the classic apple model, scald resistance is often linked to an attenuated phenylpropanoid flux, lower levels of specific CGA isomers, and higher antioxidant capacity [34]. Even in pears, studies on ‘Blanquilla’ suggested that lovastatin-induced resistance involved modulation of the phenylpropanoid and terpenoid pathways without reporting prominent alkaloid accumulation [13]. An alkaloid-dominated defense signature was identified in ‘Dangshansuli’ pear. This finding implies that in this cultivar, 1-MCP not only suppresses the damaging pathways but also actively energizes a parallel, nitrogen-containing defense system. This signature uniquely contributes to 1-MCP-induced resistance, differing from patterns observed in apples [35] and other pear varieties [39].

Beyond a mere marker of the resistant state, the physiological relevance of this alkaloid signature is strongly supported by convergent evidence for the protective functions of this class of compounds. Sun et al. [37] showed that the particular alkaloid glycine betaine reduces pericarp browning in ‘Nanguo’ pears by boosting the activities of antioxidant enzymes and proline metabolism. Glycoside alkaloid treatment enhances goji berry defense by activating key defense enzymes and remodeling the transcriptome [38].

Guided by these established protective roles, we propose a testable hypothesis for ‘Dangshansuli’ pear: the sustained accumulation of alkaloids (e.g., gramine) is a functional cornerstone of 1-MCP-induced resistance, potentially through a dual mechanism involving direct antioxidant activity and signaling-mediated fortification of the cellular antioxidant system. This specific hypothesis provides a clear focus for future multi-omics validation. Within this restructured defense framework, flavonoids likely function as a vital complementary barrier, their steady accumulation under 1-MCP (unlike in controls) implying a role in directly neutralizing ROS.

‘Dangshansuli’ pears co-express alkaloids and flavonoids in their defense profiles. Their combined presence marks a fundamental departure from phenol-centric defense mechanisms in apples. Postharvest stress elicits unique responses among various fruits, indicating that no singular metabolic defense mechanism is universally effective for all types. Consequently, our research emphasizes the necessity to understand the unique metabolic vulnerabilities and defensive mechanisms specific to each fruit cultivar. This knowledge is key to creating effective, targeted postharvest solutions.

### 4.4. Systems-Level Efficacy: Decoupling Metabolism from Stress

Integrative analyses incorporating KEGG and correlation networks offer a systemic perspective, elucidating fundamental alterations in fruit metabolic profiles induced by 1-MCP to avert disorder. Phenylpropanoid pathway serves as a key weak point in the control network. It closely links to major oxidative stress and damage indicators. Such indicators include ethylene, ROS, MDA, and PPO (Figure 6A). Tight coupling indicates superficial scald is not a localized defect but a system-wide failure, where dysregulation of core pathway is associated with propagated changes throughout the network, characterizing the disorder phenotype.

Remarkably, treatment with 1-MCP was associated with a restructuring of this network architecture. It markedly diminished the correlations between principal metabolic pathways, encompassing flavonoid biosynthesis, and the core markers of injury, which is consistent with a decreased coupling of the metabolic network to stress (Figure 6B). This shift suggests that 1-MCP’s efficacy may involve shielding the metabolic network from stress—related disruption as a whole. It does not just work by controlling how much individual pathways change. An obvious change occurs in the network configuration during stress response. The network transitions from a tightly coupled state to a more partitioned and stable arrangement. Such a change is consistent with a role for 1-MCP in enhancing the system’s inherent resilience. This systemic decoupling observed in ‘Dangshansuli’ pear—where key metabolic pathways become insulated from stress markers—may represent a generalizable principle for effective postharvest interventions. Comparing our correlation network (Figure 6B) with conceptual models from apple studies [11,34], a common theme emerges: successful scald control is associated not merely with changes in single metabolite abundance, but with a fundamental restructuring of metabolic network topology towards a more robust and less correlated state. Earlier observations in pears regarding the broad physiological impact of 1-MCP are aligned with and mechanistically explained by the finding [39]. The discovery reveals that metabolic network restructuring promotes stability.

### 4.5. Limitations and Future Perspectives

While this study provides a dynamic, severity-linked view of metabolic reprogramming by 1-MCP, our interpretation is necessarily framed by a specific comparative design: ‘pathological trajectory versus intervention-induced suppression’. This design, which contrasts the scald development pathway with the state induced by 1-MCP, is powerful for identifying metabolism critical to counteracting the disorder. However, a key interpretative consideration arises because the metabolic trajectory in control fruit inherently represents a composite of general storage-related senescence and scald-specific dysregulation. Our framework, utilizing 1-MCP-treated fruit, effectively isolates metabolic reorganization linked to disorder inhibition. To move beyond this composite view and definitively disentangle senescence from scald-specific markers, future work would require an untreated control that remains scald-free under identical conditions.

In additional, while three independent biological replicates derived from distinct orchard blocks were used, all treatments and storage were conducted within a single facility. Future studies could further strengthen the robustness of the findings by replicating the entire experiment across independent storage trials or seasons.

Notwithstanding these considerations, the severity-synchronized sampling strategy successfully captured metabolic dynamics tightly linked to the phenotypic expression and successful inhibition of the disorder. Future research should: (1) incorporate a non-scalding, untreated control to complement the current model and more precisely distinguish senescence-related metabolic shifts; (2) employ integrated transcriptomics and proteomics to validate the proposed metabolic network and uncover the upstream regulatory mechanisms, especially those governing the key alkaloid biosynthesis pathways to experimentally test the hypothesized dual roles (e.g., via exogenous application of gramine); and (3) combine targeted enzymatic assays to confirm activity changes in the predicted central metabolic pathways. Such multi-omics integration will solidify the mechanistic understanding of 1-MCP action and may reveal novel, high-precision targets for metabolism-targeted preservation strategies.

## 5. Conclusions

This study delineates the dynamic metabolic trajectory of superficial scald development in ‘Dangshansuli’ pears. The disorder progresses from an ethylene burst through escalating oxidative stress to a collapse of antioxidant defenses and irreversible membrane damage.

By directly contrasting this pathological trajectory with the metabolic state induced by 1-MCP, we demonstrate that 1-MCP treatment is associated with a comprehensive reprogramming to suppress the disorder. It functions not only as an initial ethylene antagonist but also exerts a sustained effect, involving the strategic reconfiguration of secondary metabolism. It suppresses the synthesis of scald-promoting metabolites (α-farnesene, specific phenolic acids) and concurrently is associated with the activation of the biosynthesis of protective alkaloids and flavonoids. This shift establishes a resilient metabolic state that maintains cellular homeostasis and antioxidant capacity, thereby preventing disorder development.

A defining signature of this 1-MCP-induced protected state is the sustained and coordinated accumulation of protective alkaloids and flavonoids, concurrent with the suppression of α-farnesene. Correlation network analysis further suggests that 1-MCP’s efficacy is associated with an attenuated coupling between the phenylpropanoid metabolic network and oxidative damage markers, thereby insulating key secondary metabolic pathways from stress-driven dysregulation.

Collectively, our findings suggest that 1-MCP functions not merely as a simple ethylene inhibitor but is associated with the induction of a resilient, scald-suppressive metabolic state. This work provides a mechanistic foundation for developing next-generation, metabolism-targeted preservation strategies, such as those aimed at enhancing key protective pathways like alkaloid biosynthesis.

## Data Availability

The raw mass spectrometry data, processing parameters, and sample metadata generated in this study have been deposited in the Zenodo repository and are publicly available via the permanent DOI: https://doi.org/10.5281/zenodo.18412652.

## Supplementary Materials

The following supporting information can be downloaded at: https://www.mdpi.com/article/10.3390/foods15040622/s1, Figure S1A: Hierarchical clustering analysis of differential metabolites in the control group, Figure S1B: Hierarchical clustering analysis of differential metabolites in the 1-MCP group, Table S1: The dates that all pear samples were analyzed.

## Abbreviations

The following abbreviations are used in this manuscript:

1-MCP	1-Methylcyclopropene
DPA	Diphenylamine
CTols	Conjugated trienols
PPO	Polyphenol oxidase
ROS	Reactive oxygen species
SSI	Superficial scald index
EPR	Ethylene production rate
AF	α-farnesene
MDA	Malondialdehyde
H_2_O_2_	Hydrogen peroxide
GSH	Glutathione
CGA	Chlorogenic acid
